# Quantifying uncertainty in intervention effectiveness with structured expert judgement: an application to obstetric fistula

**DOI:** 10.1136/bmjopen-2014-007233

**Published:** 2015-06-03

**Authors:** Abigail R Colson, Sweta Adhikari, Ambereen Sleemi, Ramanan Laxminarayan

**Affiliations:** 1 Center for Disease Dynamics, Economics & Policy, Washington DC, USA; 2 Princeton Environmental Institute, Princeton University, Princeton, New Jersey, USA; 3 Columbia University Mailman School of Public Health, New York, New York, USA; 4 Maimonides Medical Center, Brooklyn, New York, USA; 5 Public Health Foundation of India, New Delhi, India

**Keywords:** GYNAECOLOGY, GENITOURINARY MEDICINE

## Abstract

**Objective:**

To demonstrate a new application of structured expert judgement to assess the effectiveness of surgery to correct obstetric fistula in a low-income setting. Intervention effectiveness is a major input of evidence-informed priority setting in healthcare, but information on intervention effectiveness is generally lacking. This is particularly problematic in the context of poorly resourced healthcare settings where even efficacious interventions fail to translate into improvements in health. The few intervention effectiveness studies related to obstetric fistula treatment focus on the experience of single facilities and do not consider the impact of multiple factors that may affect health outcomes.

**Design:**

We use the classical model of structured expert judgement, a method that has been used to quantify uncertainty in the areas of engineering and environmental risk assessment when data are unavailable. Under this method, experts quantify their uncertainty about rates of long-term disability in patients with fistula following treatment in different contexts, but the information content drawn from their responses is statistically conditioned on the accuracy and informativeness of their responses to a set of calibration questions. Through this method, we develop best estimates and uncertainty bounds for the rate of disability associated with each treatment scenario and setting.

**Participants:**

Eight experts in obstetric fistula repair in low and middle income countries.

**Results:**

Estimates developed using performance weights were statistically superior to those involving a simple averaging of expert responses. The performance-weight decision maker's assessments are narrower for 9 of the 10 calibration questions and 21 of 23 variables of interest.

**Conclusions:**

We find that structured expert judgement is a viable approach to investigating the effectiveness of medical interventions where randomised controlled trials are not possible. Understanding the effectiveness of surgery performed at different types of facilities can guide programme planning to increase access to fistula treatment.

**Strengths and limitations of this study**

- This is the first application of the classical model of structured expert judgement to global health. We find it to be a feasible approach to learning about intervention effectiveness in settings where randomised controlled trials are not possible.
- Although the estimates from performance weights were statistically superior to those obtained by simply average expert responses, the uncertainty ranges for some questions were still large, limiting their usefulness.
- Future work applying structured expert judgement to questions of intervention effectiveness is needed to further refine the method.

## Introduction

Obtaining accurate estimates of intervention effectiveness is a major challenge in evidence-based global health. Intervention efficacy is best estimated through randomised controlled trials, but costs and logistical challenges make these infeasible in many instances. Randomised controlled trials may also not be possible due to ethical concerns related to not treating or providing substandard treatment to a subset of patients. Questions concerning treatment effectiveness often persist following a randomised controlled trial because such studies may have limited external validity—efficacy in randomised trials may not always translate to effectiveness under real-world health systems. Intervention efficacy does not necessarily translate from the study site and context to other settings. Efficacy studies focused on individual interventions also have limitations when applied to estimating the effectiveness of packages of interventions. The total effectiveness of a package may be less or greater than the sum effectiveness of the individual parts, but traditional efficacy studies offer no guidance on determining the effect size of the total package.

The classical model (also referred to as the ‘Cooke method’) of structured expert judgement allows analysts to use expert opinion to overcome gaps in the literature to better understand the effectiveness of an intervention or set of interventions. The method is a technique for pooling expert opinion to create rational consensus on point estimates and quantify the surrounding uncertainty.^1^ This study is the first application of the classical model of structured expert judgement to the determination of intervention effectiveness and long-term consequences. We apply the method to the question of long-term disability following treatment for obstetric fistula, in order to assess the method's applicability to quantifying uncertainty around intervention effectiveness.

Obstetric fistula is a major source of maternal morbidity in low and lower middle income countries. An estimated 3.5 million women currently suffer from fistula, with 130 000 new cases occurring every year.^2^ The 2010 Global Burden of Disease (GBD) study estimates that obstetric fistula results in 1.15 million years lived with disability (YLDs), approximately 64% of the total YLDs attributable to all maternal conditions.^3^ This high burden is despite the fact that obstetric fistula is generally preventable and treatable.

Fistula is typically a result of surgical error in high income countries, but in lower income settings fistula is largely a consequence of obstructed labour. Without access to emergency obstetric care, the internal trauma of an obstructed labour that may last several days causes tissue death in the woman's birth canal. The dead tissue sloughs away, leaving a fistula between the vagina and bladder, rectum or both. A woman with fistula may suffer from constant urinary or faecal incontinence as well as other complications caused by the internal damage. The incontinence can also lead to social and sexual ostracism, introducing psychological complications. Women with fistula are often blamed for their condition, exacerbating the social and psychological harm.

Fistula can be successfully treated with surgery. Fistula centres, such as the Addis Ababa Fistula Hospital in Ethiopia, specialise in the care and treatment of patients with fistula. Despite the high burden of disease and the existence of dedicated medical facilities, the literature on the efficacy and long-term consequences of fistula surgery is relatively sparse. Estimates of urinary incontinence, an important outcome following fistula surgery, in patients following fistula repair vary widely, from 8% to 50%.^4–8^ Recent literature reviews find that existing studies are poorly conducted, with few randomised studies, poorly defined outcome measures and most research focusing on the experience of one clinic or even one surgeon.^9–11^ Most studies also track outcomes only a couple of weeks postsurgery,^12^ ^13^ but incontinence can change over longer periods of time following discharge from the hospital.^5^ ^11^ While research examines the effect of patient and fistula characteristics on outcomes, no study explores the impact of other contextual factors, such as surgeon experience, type of hospital or whether a surgery was performed as part of a training or outreach programme.^10^ Understanding the role of these factors in determining surgery outcomes is necessary when planning programmes that expand access to fistula surgery.

However, collecting long-term outcomes data on women who underwent fistula surgery is challenging. Patients with fistula often live in remote areas, disconnected and far removed from the clinics where they sought treatment, making follow-up difficult.^5–7^ To overcome the lack of existing data sources relevant to the long-term disability of patients with fistula, we apply the classical model of structured expert judgement to explore two questions:

1. What is the long-term disability associated with different types of fistula following surgical treatment?
2. Do long-term outcomes differ for patients treated in different settings (such as a high-volume fistula centre vs a district hospital that treats a low volume of patients with fistula)?

## Methods

The classical model of structured expert judgement, so called due to its analogous use of terms from classical statistics, has been used to establish point estimates and quantify uncertainty using experts in fields including aviation, engineering, environmental health, nuclear safety and climate change.^1^ ^14^ ^15^ Whereas other forms of expert judgement—committees or the Delphi method, for example—seek to eliminate uncertainty by forcing agreement between expert participants, the classical model determines point estimates and then exploits experts’ existing uncertainty to more accurately establish CIs for the estimates. Experts are presented with a series of questions asking for potentially observable quantities, such as the rate of long-term disability, in a given population of patients with fistula. For each question, experts provide a median estimate and 90% and 50% credible ranges. The expert believes it is equally likely that the true value falls above or below the median estimate. The 90% credible range is akin to the expert's 90% CI; the expert believes there is a 90% chance that the true value falls within that range. Similarly, the 50% credible range is a narrower range, and the expert believes there is a 50% chance that the true value falls within that range. This information forms the expert's uncertainty distribution for the question. Experts’ distributions are combined in two ways: first, by assigning equal weight (EW) to all experts; and second, by assigning experts weight based on their performance.

Performance weights (PW) are determined by the experts’ assessments on a set of calibration questions for which answers are known to the study team but unknown to the experts. Experts are scored according to the statistical accuracy and informativeness of their assessments. Statistical accuracy is based on testing the hypothesis that the true values of the calibration questions (ie, the actual answers) could be jointly drawn from an expert's provided distributions. An expert with high statistical accuracy captures the true values within his or her ranges at the expected frequency. That is, as the number of calibration questions increases, the frequency of capturing the true values within the 90% credible range approaches 90%. Similarly, the frequency of capturing true values within the 50% credible range approaches 50%. Informativeness is a measure of how peaked an expert's uncertainty distributions are, with more peaked distributions indicating a narrower range of values in the credible range and thus less uncertainty. Less peaked assessments encompass a wider range of credible values—indicating more uncertainty—and thus provide less information on the indicator in question. A more detailed description of these two scores and the expert scoring procedure is available elsewhere.^1^ ^14^ ^16–19^

Calibration questions allow analysts to assess an expert's ability to accurately quantify his or her uncertainty on questions in the field of interest. Calibration questions also create the basis for identifying the optimal PW combination of experts.^14^ In this study, calibration questions focused on the epidemiology of obstetric fistula, including the prevalence of fistula in different subpopulations and rates of associated indicators of maternal well-being. Calibration questions were not designed to identify the expert most skilled at performing fistula surgery, but rather the experts able to best think about average outcomes and the surrounding uncertainty for a generic set of patients with fistula.

We identified and recruited eight experts to participate in the study. An expert fistula surgeon is someone recognised in the obstetric surgery community as a leader, trainer and highly experienced surgeon. An initial set of experts were identified by the study team, and those individuals were asked to nominate additional experts for participation. Experts were selected based on their history of working with patients with fistula in low and middle income countries. All were full-time fistula surgeons who had performed over 500 surgical cases and trained other surgeons. None of the experts were based in high income countries. Experts included a mix of urologists and gynaecologists, most with experience predominantly in Africa. Two members of the study team conducted in-person, two-on-one interviews at the International Society of Obstetric Fistula meeting held in Dhaka, Bangladesh in mid-November 2012. In addition to gathering information on experts’ uncertainty distributions for the set of questions, we asked experts to explain their thinking and provide rationales for their assessments. These narratives were captured alongside the uncertainty distribution data.

The structured expert judgement protocol included 10 calibration questions and 23 variables of interest questions (see online supplementary file). Variables of interest questions focused on five scenarios (table 1), which were constructed by the study team in collaboration with another expert in obstetric surgery. Scenarios were chosen to include a variety of factors that could impact the likelihood of successful surgical repair and were written to be specific enough that the experts’ uncertainty distributions would reflect only uncertainty in outcomes and not confusion over the clinical presentation of a given case. For each scenario, experts were asked to predict how many patients, given 1000 such cases, would develop long-term disability if treated in one of three settings (a hospital in a high income country, a high-volume fistula centre in a low income country, and a low-volume district hospital in a low income country) or if left untreated. For scenario 3, experts were also asked how many of 1000 such cases would result in death if treated in the different settings.

**Table 1:** Scenarios described in variables of interest questions

Scenario	Case description
1	An 18-year-old female patient had obstructed labour and delivery of a stillbirth 1 month ago. She has a large vesicovaginal fistula that obliterated the anterior vaginal wall, resulting in total loss of the urethra. On examination, she has involvement of both ureters, with partial obstruction of one. The main long-term complications are the constant leakage of urine (urinary incontinence) and functional loss of a kidney
2	A 22-year-old female patient presents with urinary incontinence after having a stillbirth. She has a small fistula but bilateral foot drop. Long-term disability includes urinary incontinence with severe hip and leg pain, difficulty in mobility and atrophy of the lower extremities
3	A 27-year-old female patient presents after labouring for 3 days in her village during her 3rd pregnancy. She undergoes an emergency caesarean delivery of a stillbirth. Possible long-term outcomes include: loss of her uterus leading to infertility, and damage to the bladder and/or ureters due to the difficult nature of the surgery
4	A 17-year-old female patient presents in labour with obstruction for the last 24 h. She delivers a live infant who has some transient respiratory depression, but otherwise appears well. She develops a rectovaginal fistula on post partum day 1. Potential long-term disability includes severe faecal incontinence, imposed isolation from her family and depression (For this scenario, we asked experts about both rates of long-term disability and death)
5	A 30-year-old woman underwent an emergency caesarean delivery after presenting to a hospital with severe antepartum haemorrhage at term. Her recovery is complicated by the constant leakage of urine during her postoperative course, despite placement of a Foley catheter for drainage. She is diagnosed with a right-sided ureterovaginal fistula. Potential long-term disability is severe urinary incontinence, depression, isolation and the possible loss of kidney function

Questions asked about the rate of long-term disability and, for scenario 3, the rate of death per 1000 cases treated at different facility types or untreated.

## Results

**Figure 1:**
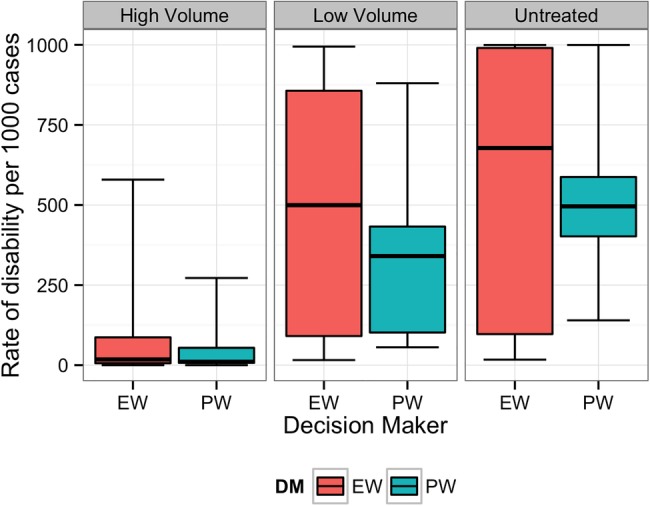
Equal-weight (EW) and performance-weight (PW) decision maker (DM) results on the rate of long-term disability for scenario 1. Note: For all figures, boxplots indicate the 5th, 25th, 50th, 75th and 95th centile values from the given expert's or DM's uncertainty distributions.

**Figure 2:**
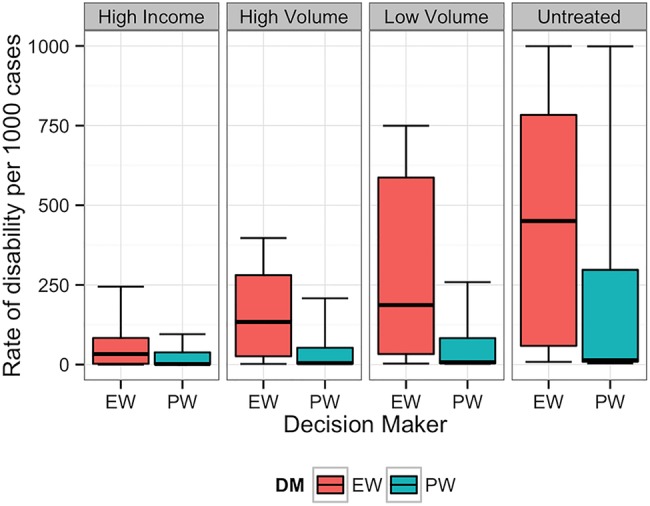
Equal-weight (EW) and performance-weight (PW) decision maker (DM) results on the rate of long-term disability for scenario 2. Note: For all figures, boxplots indicate the 5th, 25th, 50th, 75th and 95th centile values from the given expert's or DM's uncertainty distributions.

**Figure 3:**
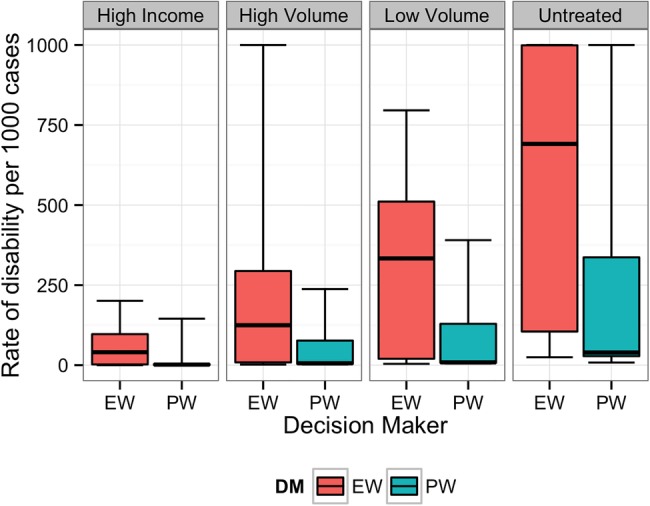
Equal-weight (EW) and performance-weight (PW) decision maker (DM) results on the rate of long-term disability for scenario 3. Note: For all figures, boxplots indicate the 5th, 25th, 50th, 75th and 95th centile values from the given expert's or DM's uncertainty distributions.

**Figure 4:**
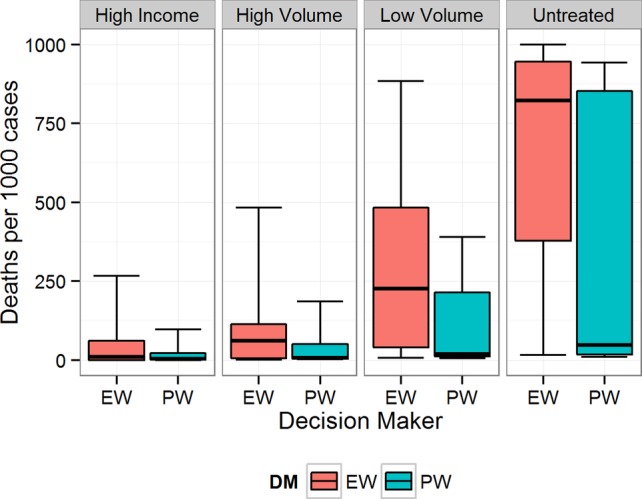
Equal-weight (EW) and performance-weight (PW) decision maker (DM) results on the rate of death for scenario 3. Note: For all figures, boxplots indicate the 5th, 25th, 50th, 75th and 95th centile values from the given expert's or DM's uncertainty distributions.

**Figure 5:**
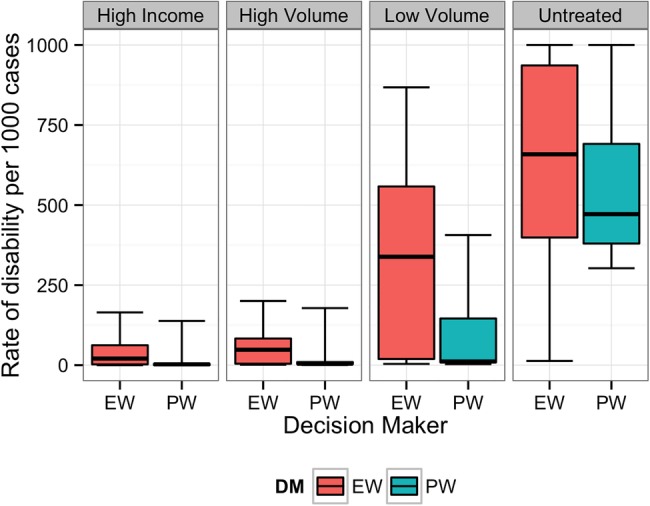
Equal-weight (EW) and performance-weight (PW) decision maker (DM) results on the rate of long-term disability for scenario 4. Note: For all figures, boxplots indicate the 5th, 25th, 50th, 75th and 95th centile values from the given expert's or DM's uncertainty distributions.

**Figure 6:**
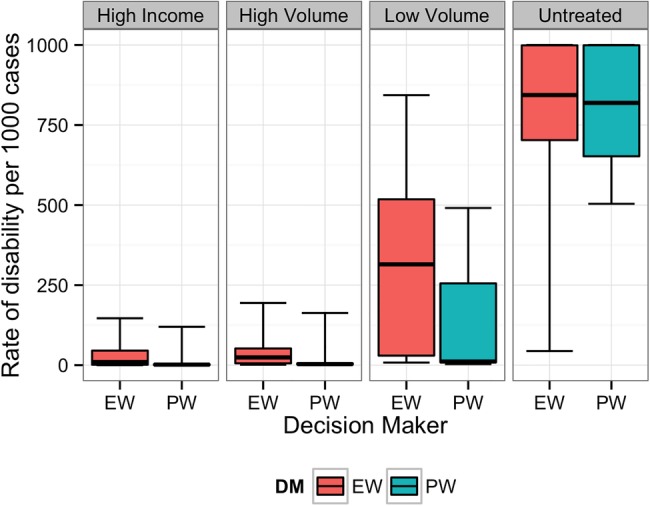
Equal-weight (EW) and performance-weight (PW) decision maker (DM) results on the rate of long-term disability for scenario 5. Note: For all figures, boxplots indicate the 5th, 25th, 50th, 75th and 95th centile values from the given expert's or DM's uncertainty distributions.

Figures 1–6 show the EW and PW pooled combinations of experts, referred to as decision makers (DMs), for each variable of interest. The median estimate of the PWDM is lower than that of the EWDM for all 23 variables of interest, indicating lower rates of long-term disability and death for each scenario following any treatment or if untreated. The PWDM's 50% credible ranges are narrower than those of the EWDM for all 10 calibration questions and 21 of the 23 variables of interest. The PWDM's 90% credible ranges are narrower for 9 of the 10 calibration questions and 21 of 23 variables of interest. This is the benefit of the classical model of structured expert judgement: PW typically yield pooled assessments that are more informative than the EW combination while remaining at least as statistically accurate.^14^

The DMs indicated that experts were most certain about long-term outcomes when patients received treatment in high income countries or high-volume fistula centres. Uncertainty increased when experts thought about outcomes following treatment in low-volume district hospitals or if patients did not receive treatment.

**Figure 7:**
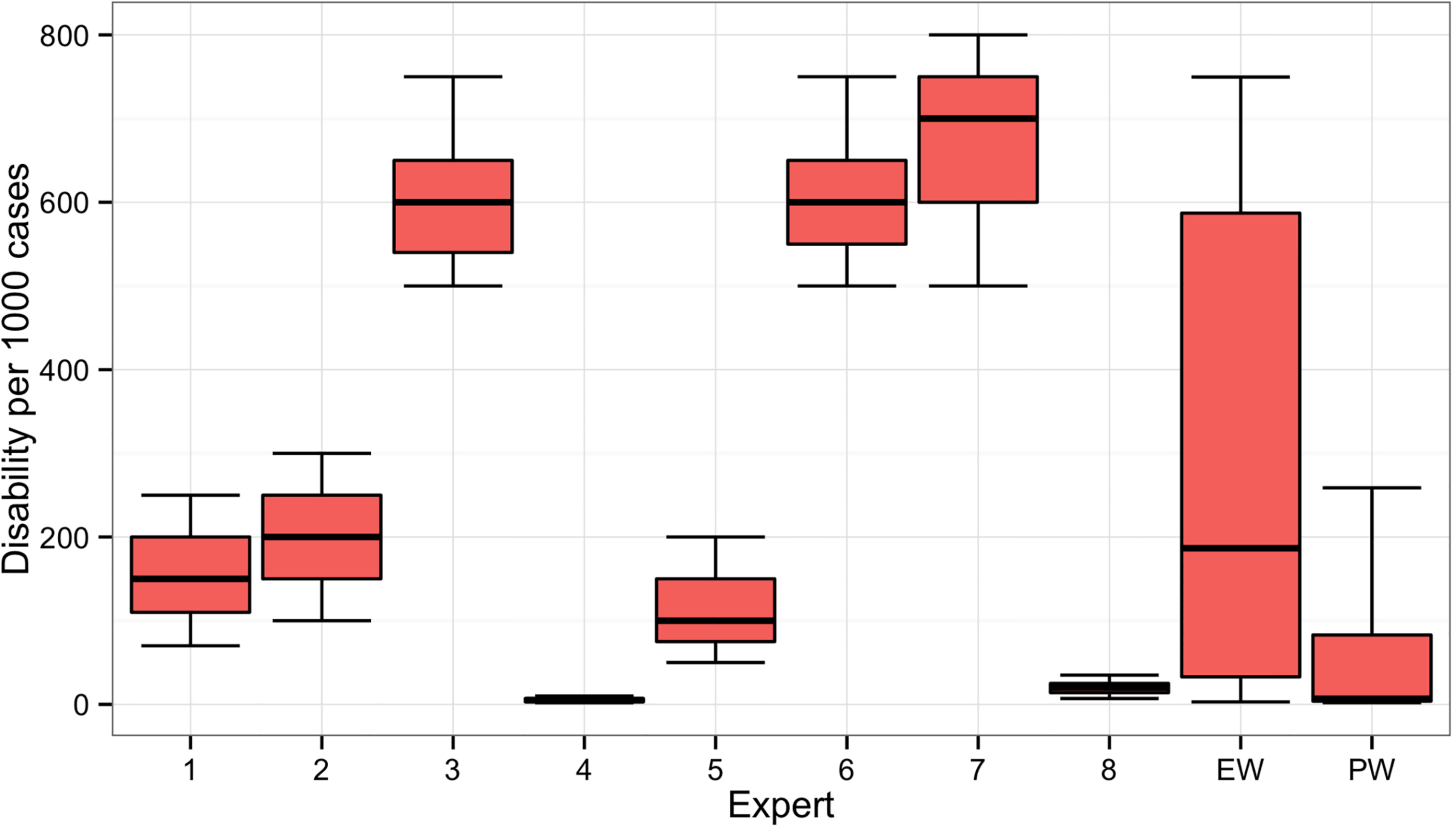
Individual expert and pooled decision maker assessments for scenario 2 for patients treated in a low-volume hospital. Note: Results are shown for each individual expert (1–8), the equal-weight (EW) decision maker and the performance-weight (PW) decision maker. For all figures, boxplots indicate the 5th, 25th, 50th, 75th and 95th centile values from the given expert's or decision maker's uncertainty distributions.

Experts disagreed on the likelihood of long-term disability for women suffering from fistula who receive treatment in low-volume facilities or do not receive any treatment. For example, figure 7 shows the individual expert responses and pooled DMs for the question asking about scenario 2 patients treated in a low-volume district hospital. The experts’ assessments reflected two divergent viewpoints on the long-term outcomes. One group believed that even in a low-volume district hospital, facilities will have the resources necessary to adequately treat the patient, and thus long-term disability will not be common. A second group of experts were of the opinion that district hospital doctors would be too unskilled to prevent long-term disability in these patients.

## Discussion

In this study, the PWDM indicates lower rates of disability and death for each scenario and treatment facility, including no treatment, than the EWDM. The PWDM also shows less uncertainty around its median estimates than the EWDM. Uncertainty increases, however, when experts shift from thinking about outcomes in high-volume centres to outcomes in low-volume centres or among untreated women with fistula. The experts we interviewed work predominantly in high-volume fistula centres. Existing short-term outcome research also focuses on patients who receive care at fistula centres.^10^ ^12^ Experts are most familiar with outcomes for patients in this setting, and this is reflected in the narrower uncertainty ranges given for patients treated in high-volume fistula centres. In contrast, the experts we interviewed do not see as many patients in low-volume district hospitals and, by definition, do not see any untreated women with fistula. Data on short-term and long-term outcomes in these settings do not exist. Thus, the experts’ assessments show greater uncertainty when considering long-term outcomes in these settings.

Experts expressed a great deal of uncertainty about long-term outcomes for patients who were not treated. Differences in the median PWDM estimates for untreated patients across the scenarios, however, suggest that some untreated fistulas are more problematic than others. The low median values for untreated patients in scenarios 2 and 3 indicate that experts thought those cases could resolve even if untreated, whereas that was much less likely for the other scenarios.

In all scenarios, the PWDM signifies that the rate of long-term disability or death is low for patients treated in high-volume fistula centres. The PWDM also shows low rates of disability or death for patients treated in low-volume district hospitals, but the higher upper bounds on the 50% and 90% credible ranges indicate that experts are less certain about outcomes following treatment in this setting. Only one scenario, scenario 1, has a PWDM median rate of disability substantially higher in low-volume versus high-volume hospitals, indicating that this is a more complicated case that experts know benefits from specialised care. The PWDM for scenario 2, in contrast, shows little difference between the ranges of rates for high-volume and low-volume settings, suggesting that this case may be easier to repair. This information can inform future fistula treatment programmes. Helping district hospitals identify and treat relatively simple cases like this can improve the quality of fistula care in a region. For more complicated cases, however, where median rates of disability are higher (eg, scenario 1) or the uncertainty about outcomes is much larger (eg, scenarios 3–5), the best treatment strategy may be to refer patients to a high-volume centre for the first repair. The difference between expected outcomes following treatment in a high-volume specialty hospital rather than a low-volume district hospital confirms the valuable role specialty care needs to continue to play in a comprehensive fistula treatment programme.

Estimates of disability and mortality following fistula repair from our study are not directly comparable to the existing literature for several reasons. First, our study focused on five specific scenarios of fistula rather than all cases generally. These scenarios may not represent the full range of cases seen in an observational study. However, the specific cases presenting under our scenarios will vary somewhat. Experts were asked to fold these expected variations into their uncertainty ranges. In an observational study, however, some patients may be dropped under the study's inclusion criteria. Second, all of our cases focus on the first attempt to repair fistula. Surgery outcomes worsen if patients have a history of previously unsuccessful repair, and observational studies based on patients at a single fistula centre may include a high proportion of patients referred from other facilities after a previously failed repair attempt.^11^ Third, continence may improve over time, and few studies look at long-term repair outcomes, due to the challenge of off-site follow-up.^5^ ^11^ Thus, existing studies may under-report the actual rate of no incontinence following fistula repair.

Despite these challenges, it is still instructive to compare our results with those from other studies. A recent review by Arrowsmith and colleagues of the existing literature on fistula surgery success rates identifies reported closure rates of 53–97.5%, with an average of successful closure in 86% of patients. Defining success as ‘no incontinence’ rather than closure lowers the reported success rates. The average of reported rates of no incontinence is 70%, with a range of 42–92%.^11^ Experts in our study were instructed to consider incontinence as a potential long-term disability following fistula repair surgery. The estimated surgery success rates across the five scenarios in this study are higher than those identified in existing research by Arrowsmith and colleagues. Here, the PWDM identifies median surgery success rates, based on our definition of long-term disability, at between 98.9% and 99.7% in our scenarios for patients treated in a high-volume fistula hospital. Lower bounds of success (based on the given 95th centile rates of disability) range from 72.8% to 83.7%, and upper bounds (based on the 5th centile rates of disability) are all near 100%.

We are unable to compare our results with those from GBD 2010 because GBD disability weights sum the impact of all fistulas, whereas we estimate outcomes within certain subsets of fistula. Without knowledge of the frequency with which these scenarios occur and the rates at which patients seek treatment for fistula, it is not possible or reasonable to translate our estimates into a single disability weight. Although structured expert judgment can be a useful method for estimating disability weights, our objective in this study was slightly different. Rather than quantify the total disability attributable to obstetric fistula, we aimed to estimate the rate of disability associated with untreated and treated fistula, to better understand the effectiveness of fistula surgery.

Structured expert judgement provides estimates and uncertainty ranges for rates of long-term disability and death in a variety of treatment contexts. Such estimates would not be possible with existing data sources, and collecting the necessary data in the future would be difficult, unethical and/or expensive. Structured expert judgement also presents a new opportunity to estimate long-term success rates in context when long-term outcomes are difficult to track. The classical model of structured expert judgement presents a new opportunity to study questions of efficacy and effectiveness where these data limitations exist.
